# Effects of Commercial Arbuscular Mycorrhizal Inoculants on Plant Productivity and Intra-Radical Colonization in Native Grassland: Unintentional De-Coupling of a Symbiosis?

**DOI:** 10.3390/plants11172276

**Published:** 2022-08-31

**Authors:** Eric B. Duell, Adam B. Cobb, Gail W. T. Wilson

**Affiliations:** 1 Kansas Biological Survey and Center for Ecological Research, Lawrence, KS 66047, USA; 2 Soil Food Web School, LLC, Corvallis, OR 97330, USA; 3 Department of Natural Resource Ecology and Management, Oklahoma State University, Stillwater, OK 74075, USA

**Keywords:** arbuscular mycorrhizal (AM) fungi, commercial inoculum, grasslands, restoration, symbiosis

## Abstract

There has been a surge in industries built on the production of arbuscular mycorrhizal (AM) fungal-based inoculants in the past few decades. This is not surprising, given the positive effects of AM fungi on plant growth and nutritional status. However, there is growing concern regarding the quality and efficacy of commercial inoculants. To assess the potential benefits and negative consequences of commercial AM fungal inoculants in grasslands, we conducted a controlled growth chamber study assessing the productivity and AM fungal root colonization of nine grassland plant species grown in grassland soil with or without one of six commercial AM fungal products. Our research showed no evidence of benefit; commercial inoculants never increased native plant biomass, although several inoculants decreased the growth of native species and increased the growth of invasive plant species. In addition, two commercial products contained excessive levels of phosphorus or nitrogen and consistently reduced AM fungal root colonization, indicating an unintentional de-coupling of the symbiosis. As there is little knowledge of the ecological consequences of inoculation with commercial AM fungal products, it is critical for restoration practitioners, scientists, and native plant growers to assess the presence of local AM fungal communities before investing in unnecessary, or possibly detrimental, AM fungal products.

## 1. Introduction

Rising global human populations with their corresponding food demands, combined with increased environmental concerns, including reductions in dependency on energy-intensive agrochemicals [1], have led to a demand for sustainable agriculture that includes a remarkable surge in the development of commercial biofertilizers. There is also potential for economic value, as it is estimated that the agricultural biofertilizer market for microbial inoculants is likely to reach USD 2.3 billion by 2022 [2] and is expected to reach USD 11.45 billion by 2026 [3]. Arbuscular mycorrhizal (AM) fungi are increasingly common in biofertilizer production with an unprecedented boom of the mycorrhizal inoculant industry [4,5,6]; this is not surprising, as many results from greenhouse and field trials have shown positive effects of AM fungal inoculation. AM fungi, which colonize roots and provide nutrients in exchange for photosynthates, establish symbiotic relationships with more than 85% of plant species, including most crop species, and are considered to play an important role in natural and agricultural systems [7]. Numerous studies have shown that inoculation with AM fungi, especially in low pH soils, could improve plant growth [8,9,10,11,12] and may increase plant resistance to pathogens [13] and other abiotic stresses, such as drought or salinity [14]. At the ecosystem level, AM fungi have been shown to improve soil structure [15], increase soil C storage [16], and reduce soil nutrient loss [17].

However, there is growing concern regarding the lack of mandatory quality control of commercial AM fungal inoculants. While other bioinoculants, such as rhizobia, have strong and consistent research showing benefits following inoculation, there has, to date, been little evidence that inoculation with commercial AM fungi is beneficial, even in cropping systems with low AM inoculum potential [2,5,18]. In fact, previous studies consistently show that ineffective AM fungal inoculants are extremely common [5,19,20,21]. For example, in a recent controlled study examining the effectiveness of 28 commercial AM inoculants in non-sterile soil, no increase in AM fungal root colonization was observed, and only one inoculant increased plant biomass [20]. Additionally, when assessing plants grown in sterile soil, 84% of the AM inoculants did not lead to AM fungal root colonization, indicating a lack of viable propagules present in these products [20].

The majority of AM biofertilizer production has targeted horticulture and field crop production, especially with the focus of increasing cereal production [2], and these agricultural soils typically contain low AM inoculum potential. Assessing the effects of commercial inoculum under these conditions likely increases the probability of detecting a positive biofertilizer inoculation outcome as plants are expected to respond positively to inoculation when growing in an inoculum-limited environment [22], as propagule abundance is tightly linked to mycorrhizal response [23]. However, there is growing interest among pasture and rangeland producers to utilize commercial AM fungal inoculants to increase forage quality for livestock production [18]. Compared to horticulture and field crop production, most grasslands contain diverse, native, AM fungal propagules, and little research has been conducted on the influence of commercial AM fungal inoculants when native AM fungi are already present [18]. While studies in degraded systems are a useful indication of commercial inoculant performance in extreme conditions, they cannot inform inoculant use in systems where resident AM fungal communities are present. To balance our understanding of inoculant performance, it is important to conduct inoculation experiments across a broad range of soil conditions, including intact ecosystems such as native grasslands, to better predict where inoculation would be effective.

Our study compares the above- and belowground productivity and AM fungal colonization of nine grassland plant species, including species that are native or invasive to the central Great Plains grasslands of North America. Plants were grown in local grassland soil (containing indigenous AM fungi) or local soil containing native AM fungi combined with one of six commercial AM products. Our research helps elucidate potential outcomes, including potentially unintended and negative consequences, of using commercial AM inoculants in grasslands.

## 2. Materials and Methods

### 2.1. Soil Preparation

Native grassland soil, Renfrow/Grainola (eroded silty clay Mollisol/Alﬁsol), was collected from the Oklahoma State University Range Research Station (pH = 6.6, plant-available N = 23.5 g kg^−1^, plant-available P = 9 g kg^−1^, K = 146 g kg^−1^, OM = 2.7%). The Soil, Water, and Forage Analytical Laboratory (SWAFL) at Oklahoma State University analyzed the baseline soil samples. Soil NO3-N and NH_4_ were extracted using a 1 M KCl solution and analyzed using the Lachat Quickchem 8000 Flow Injection Autoanalyzer [24]. Two grams of soil were extracted with 20 mL Mehlich 3 solution [25] for plant-available P and K, and the concentrations of P and K in the extract were measured using inductively coupled plasma emission spectroscopy (ICP) [26]. Soil pH was measured using a pH electrode in a 1:1 soil-to-water suspension. Soil organic matter (SOM) was determined with dry combustion using the LECO Truspec CN analyzer [27]. A total of 462 plastic pots (6 cm dia × 25 cm depth; Stuewe & Sons Inc., Tangent, OR, USA) was each filled with 600 g of freshly collected soil.

### 2.2. Seedling Establishment

We selected nine grassland plant species that included two native warm-season grasses; one non-native warm-season grass; one native and one non-native cool-season grass; and four native forb species (Table 1). The plant species used in the experiment are common to the grasslands of the central US. Seeds were germinated in vermiculite and were maintained in an 18–22 °C greenhouse. Three weeks after grass emergence and ten weeks after forb emergence, seedlings were transplanted into pots, with one seedling per pot. As these perennial species were established from seeds rather than perennating organs, the slower germination and seedling growth rates of the forbs required a longer seedling establishment period prior to their transplantation into pots.

### 2.3. Commercial Inoculum Treatments

Six replicates of each plant species were transplanted into the native grassland soil containing native AM fungal spores (Table 2). Six additional replicates of each plant species were inoculated with 5 g of one of six commercial AM inoculants added to seedling rhizospheres at transplant. Each commercial inoculant contained AM fungal spores, as indicated by the product label information (Table 2). All products used in the study came in the form of granular inoculum and were applied at recommended rates. Three products used in the study did not report a propagule type, while the remaining three specified spores as the propagules. Products A and C also contained 7 species of ectomycorrhizal fungi. Inoculum pH and plant-available N and P were determined by the SWAFL at Oklahoma State University (Table 3). Inoculant sources were de-identified for the purpose of this study [22]. Plants were watered daily and maintained in a growth chamber for 9 weeks under environmental conditions, similar to the growing conditions of selected plant species during the growing season (24–29 °C; 12:8 light:dark period). Plants were randomized in a complete block design with six replications.

### 2.4. Data Collection

After 9 weeks, plants were harvested, and roots were washed free of soil over a 2 mm sieve. Harvested biomass was oven-dried for 48 hours at 60 °C, at which time shoots, roots, and total biomass were recorded. Sub-samples of dried roots were stained with trypan blue in lacto-glycerol [28] and measured under a digital microscope (Hirox KH 7700, Tokyo, Japan) using a magnified gridline intersect method [29]. To determine the percentage of AM fungal root colonization, all observed AM fungal structures (intra-radical hyphae, arbuscules, vesicles, and coils) observed from three random sections of root lengths were scored separately, with a total of 150–300 grids observed per sample [30]. Reported colonization values of each sample were a mean of these three sub-samples.

### 2.5. Statistical Analyses

Prior to analyses, all data were tested for normality and homogeneity of variances, using Shapiro–Wilk and Levene’s tests, respectively. To test for differences in plant-available nitrogen and phosphorus of commercial inoculum products, a one-way analysis of variance (ANOVA) was employed, with inoculum identity as the independent variable. A post hoc Tukey’s honest significant difference (HSD) test was then conducted for all pairwise comparisons. Similarly, AM fungal root colonization and total biomass were analyzed using a one-way analysis of variance, with inoculum as the independent variable. Shoot and root dry masses were highly correlated with total dry mass for all plant species; therefore, only dry masses were included for simplification of data presentation. To test for differences among inocula within each plant species, post hoc Tukey’s HSD tests were conducted, with significance set at α = 0.05. Lastly, for each plant species by inoculum combination, effect size (Cohen’s d) was calculated using the function “cohen.d” in the R package effsize [31]. All analyses were conducted in R version 4.1.0 [32].

## 3. Results

### 3.1. Plant Biomass Production

**Figure 1:**
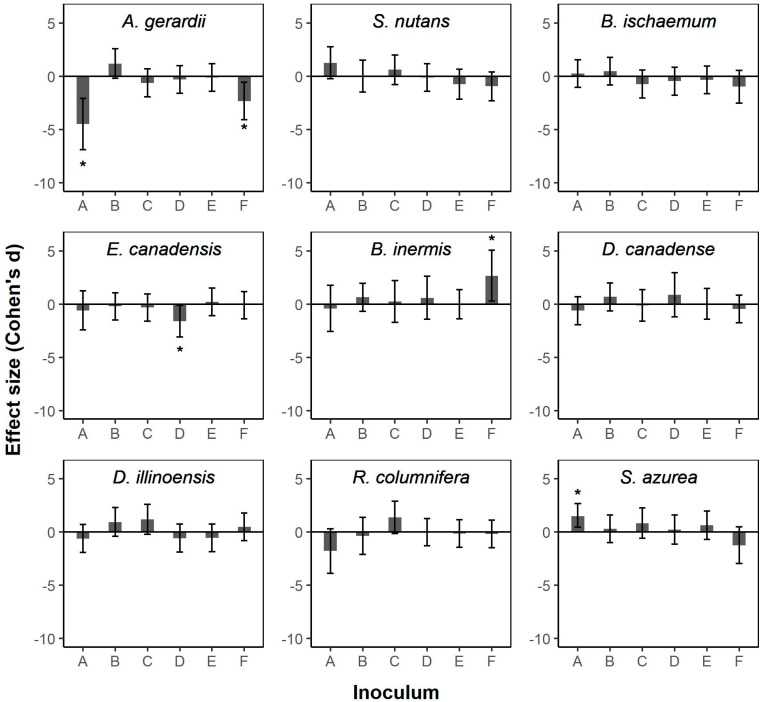
Effect sizes (Cohen’s d) of commercial arbuscular mycorrhizal (AM) fungal inoculants on total biomass productions of nine plant species commonly found in central North American grasslands (*Andropogon gerardii*; *Sorghastrum nutans*; *Bothriochloa ischaemum* (invasive); *Elymus canadensis*; *Bromus inermis* (invasive); *Desmodium canadense*; *Desmanthus illinoensis*; *Ratibida columnifera*; *Salvia azurea*). Descriptions of inoculums A–F are given in Table 2. Error bars represent 95% confidence intervals. Asterisks indicate significant effect sizes, as determined when confidence intervals did not overlap zero.

Across all of the plant species in our study, there were few significant responses in biomass production following inoculation with commercial AM fungal products. The biomass production of Andropogon gerardii, a native warm-season perennial grass species, was significantly reduced following inoculation with commercial product A (*d* = −4.48, 95% CI: −6.89 to −2.07) or product F (d = −2.32, −4.088 to −0.551) (Figure 1; Table S1). The biomass production of *Elymus canadensis*, a native cool-season perennial grass species, exhibited significant negative growth responses to commercial product D (*d* = −1.62, 95% CI: −3.097 to −0.134) (Figure 1; Table S1). A non-native invasive cool-season perennial grass, *Bromus inermis*, displayed a significant positive growth response to product F (*d* = 2.66, 95% CI: 0.29–5.040 (Figure 1; Table S1), and *Salvia azurea*, a common native perennial forb species, exhibited a positive growth response to product A (*d* = 1.50, 95% CI: 0.047–2.69).

### 3.2. AM Fungal Root Colonization

**Figure 2:**
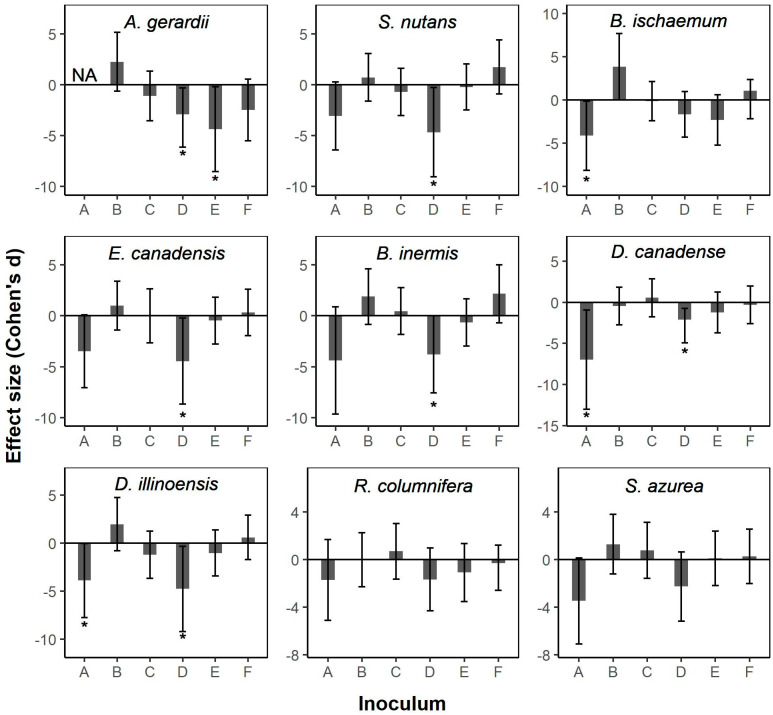
Effect sizes (Cohen’s d) of commercial arbuscular mycorrhizal (AM) fungal inoculants on the intra-radical colonization of nine plant species commonly found in central North American grasslands (*Andropogon gerardii*; *Sorghastrum nutans*; *Bothriochloa ischaemum*; *Elymus canadensis*; *Bromus inermis* (invasive); *Desmodium canadense*; *Desmanthus illinoensis*; *Ratibida columnifera*; *Salvia azurea*). Descriptions of inoculums A–F are given in Table 2. Error bars represent 95% confidence intervals. Asterisks indicate significant effect sizes, as determined when confidence intervals did not overlap zero. Note: Due to high mortality of *A. gerardii* with product A, too few root samples were collected to assess AM fungal colonization; represented here as NA.

Intra-radical AM fungal colonization exhibited negative responses to at least one commercial AM fungal product in seven of the nine plant species used in this experiment (Figure 2; Table S2). Six of these seven species (*A. gerardii, S. nutans, E. canadensis, B. inermis, D. canadense*, and *D. illinoensis*) displayed negative AM fungal colonization responses to inoculum D, while the root colonization of three species (*B. ischaemum, D. canadense*, and *D. illinoensis*) was negatively affected by product A (Figure 2; Table S2).

### 3.3. Inoculum Nutrient Content

Overall, there were significant differences in nutrient content among the different commercial inoculum products. For example, plant-available nitrogen varied widely, from 108 g kg^−1^ in inoculum A to just 3 g kg^−1^ in inoculum E (F5,12 = 69.47, *p* < 0.0001; Table 3). Similarly, plant-available phosphorus ranged from 1099 g kg-1 in inoculum A to 27 g kg^−1^ in inoculum B (F5,12 = 520.6, *p* < 0.0001; Table 3).

## 4. Discussion

Although each of the plant species used in our study was previously shown to be responsive to AM fungal symbioses [33], inoculation with commercial AM fungal products increased the productivity of only one non-native cool-season grass (*B. inermis*) and one native forb (*S. azurea*), compared to the non-inoculated control plants. Further, these increases in production occurred with only product A or product F, and all other products resulted in no increases in biomass. The lack of improved biomass production indicated that no apparent additional benefits were conferred when inoculating plants growing in soils already containing a relatively diverse local AM fungal community. Furthermore, one native warm-season grass (*A. gerardii*) and one native cool-season grass (*E. canadensis*) produced significantly less biomass following inoculation with commercial product A or F, or product D, respectively, indicating that the added expense of inoculum application could result in adverse effects, as opposed to the promotion of plant production.

The assessment of plant biomass production is the most common metric for determining the benefits of commercial inocula. This focus is understandable given that increasing aboveground production while reducing cost is typically the ultimate goal of sustainable practices. However, monitoring plant growth alone is not sufficient to properly evaluate the long-term impacts of inoculation or assess long-term agricultural sustainability; we must also consider how commercial inoculants influence mycorrhizal symbiosis for optimal growth and production. An important metric to assess the status of mycorrhizal symbiosis is the relative abundance of AM fungi colonizing the host plant root system. Although colonization is not always tightly correlated with conferred host plant benefits [34], it remains one of the standard proxies for the strength of the plant–AM fungal relationship [35]. In our study, the AM fungal root colonization of seven of the nine plant species was significantly reduced following inoculation with at least one of the commercial products, compared to colonization when host plants were grown in native soil without commercial inoculum. Of these seven species, significant negative responses were consistently found for inoculum A or D, or both, with one native plant species (*A. gerardii*) negatively responding to product E. Notably, none of the commercial products we tested increased AM fungal root colonization following inoculation. Previous research also reported lower intra-radical colonization in commercially inoculated field soils, relative to non-inoculated field soils [36], suggesting a de-coupling of the plant–fungal symbiosis. Loss of AM hypha from soils could have serious consequences at the ecosystem level, as AM fungi play a critical role in the formation of soil structures [37,38] and enhance soil C storage because they transfer C away from root surfaces, where microbial metabolism is the greatest, into the soil matrix, including aggregates [15,39]. The abundance of AM fungi is a dominant factor in soil aggregation [16], an ecosystem-level variable that influences virtually all nutrient cycling processes and soil biota [40]. Therefore, the potential to de-couple the plant-AM fungal symbiosis with concomitant losses in hyphal abundance is an important factor to consider when selecting commercial AM fungal-based inoculants; there are serious ecosystem-level consequences to the loss of AM fungi.

The observed changes (positive or negative) following the addition of commercial inoculants may be due to “fertilizer effects” rather than effects obtained from AM fungi, as the inclusion of nutrients with the inoculant carrier is common in commercial inoculants [2,18]. It has been well established that soil fertility modulates plant–AM fungal relationships, and mycorrhizal symbioses are often the most beneficial in phosphorus-limited systems, such as grasslands [41], as the primary role of AM fungi is the uptake and transport of limiting soil nutrients, such as phosphorus or nitrogen. If otherwise limiting nutrients are readily available due to fertilization, the need for AM fungal partnerships is diminished. In our study, consistent negative responses of AM fungal colonization occurred when plants were inoculated with either product A or product D. Product A contained plant-available phosphorus levels at nearly 1100 g kg^−1^ and product D contained >300 g kg^−1^ of phosphorus. Sylvia et al. [42] observed that plant benefits from AM fungi were most apparent in soils with less than 10 g kg^−1^ of P, while the mycorrhizal responsiveness of warm-season grasses grown in soils with a phosphorus availability of >40 g kg^−1^ resulted in a substantial loss of AM-derived benefits [43]. In addition to reductions in AM fungal abundance, caution is required, as extremely high nutrient concentrations can be harmful and cause plant mortality [44]. It is highly concerning that concentrations of plant-available nitrogen or phosphorus were not identiﬁed on the product labels used in our study, nor on the 28 commercial inoculants assessed by Salomon et al. [20].

Manufactured and commercialized AM fungal inoculants typically include either a single AM fungal species or mixtures of species that may or may not be native and are typically not co-adapted to the sites being inoculated. Inoculation with non-native AM fungal propagules may have serious consequences for natural systems, particularly for indigenous fungal and plant communities. In our study, *Rhizophagus irregularis* was included as a component, or as the only, fungal species of five of the six products. However, *R. irregularis* was not present in the native grassland soil. Commercial AM fungal products often contain one or two “weedy” fungal species that offer little, if any, benefit to mid- and late-successional plant species [45]. In a study assessing 68 mycorrhizal products, Basiru et al. [2] found that 100% of the products included only species from Glomeraceae, of which *R. irregularis* was contained in 39% of the products. Commercial fungi are subjected to intense breeding pressure [46], potentially leading to the selection of highly competitive traits, such as high sporulation and a propensity to invest in their own reproduction rather than in their mutualistic relationships with host plants [47,48,49]. In fact, competitiveness has been seen as a desirable trait in fungal inoculants, as it increases establishment success [50,51]. However, this is concerning, as competitive inoculants with lower mutualist quality may be more likely to become invasive [52]. It is still unclear how resident fungal communities respond to the introduction of a novel fungal species [53,54], and inoculation may have little or no impact on the resident fungal community [55]. However, there is consistent agreement that inoculation with non-native AM fungal propagules can partially replace indigenous AM fungal communities [56,57] or native AM fungal taxa may be completely replaced if the introduced species are better adapted to local niche requirements, typically with a concomitant decrease in plant productivity [8,20,49,56,57]. Although there is considerable concern that inoculation with a novel species or genotype that is not locally co-adapted may result in population-level changes, there is a lack of data on the potential long-term alterations to native AM fungal communities following inoculation with commercial bioinoculants. If these changes in AM fungal communities result in functional changes, such as losses in soil aggregation, alterations in nutrient uptake, or shifts in soil microbial biodiversity, ecosystem functioning may also be adversely affected [52,54,58,59,60].

While previous and ongoing research highlights the potential risks of commercial AM inoculum, the most urgent need is for the establishment of global quality standards, which are clearly currently lacking [5,6,18,19,20,53,61]. Although regulating the quality of commercial biofertilizers is just recently being considered, the use of locally collected and adapted plant seed has long been the recommended protocol for the restoration of disturbed ecosystems [62]. Federal regulations are in place in the United States to control the shipment of seeds, prohibit the shipment of noxious species, and require specific labels indicating the species contained in each product [63], but no oversight is currently being considered for the regulation of biofertilizer products in most countries. However, quality control mechanisms to regulate AM fungal inoculants were established in Japan with the Soil Productivity Improvement Act in 1996 [64], and a recent legislature included biofertilizers in EU fertilizer regulations (effective from April 2019), establishing standard methods for the product certiﬁcation of AM fungi inoculants [5]. Building on these efforts in Japan and the EU, Salomon et al. [5] proposed essential quality criteria and quality control measurements to be met by bioinoculant producers. These criteria could be used to improve the adoption and success of AM fungal inoculants, as previous and ongoing research, including our current study, demonstrates that there is an urgent need for the establishment of global quality standards for AM fungal biofertilizers.

Establishing a regimented control over quality and product control of AM fungal bioinoculants is essential, as it has long been established that the reintroduction of AM fungi could be critical to the re-establishment of plant species in areas with a land use history, where the soil community is drastically altered by disturbances, such as the reclamation of mine spoils [65], abandoned agriculture [46], or recovery from invasive plant species [66]. Previous studies have shown that additions of whole soil from adjacent native ecosystems, including native AM fungal communities, are key to the establishment of native plant species [67,68,69,70,71,72]. However, acquiring soil from native ecosystems is not practical for large-scale restorations, as the collection and transfer of enough native topsoil are highly destructive to the very ecosystems we are trying to restore and protect, and these soils may not be available. To address this challenge while promoting the establishment of diverse and native AM fungal communities, improved methods in AM bioinoculants are currently being developed, and there is much promise regarding the culturing of beneficial microbiota for grassland restorations [45,66,73]. However, locally adapted, native inocula are currently only commercially available for grassland ecosystems. In our current study, only one commercial inoculum, product C, contained a suite of fungal species similar to those isolated from the native soil and was also the only commercial product that did not contain *R. irregularis*.

As outlined by Salomon et al. [5] and Koziol et al. [45], innovative partnerships and improved communication between inoculum companies, regulatory agencies, scientists, primary producers, and restoration practitioners are critical for ensuring beneficial biofertilizers. In addition, the selection of AM inoculants must be appropriate for the desired application scenario, and land use history should be included in the decision to invest in commercial biofertilizer products. Our research showed that areas with low disturbances, such as intact grasslands, did not receive benefits from AM biofertilizers. Prior to the inoculation with commercial products, it should be determined if any increases in production or yield are sufficient to offset the added expense of inoculum application as many commercial products are not beneficial to production or restoration success. Furthermore, potential adverse ecological consequences following the use of AM fungal biofertilizers are not fully determined, and the ethical and economic consequences may be profound, ranging from customer quality assurance to soil biodiversity loss or overall ecosystem functioning.

## 5. Conclusions

Our work highlighted largely unexplored risks associated with the addition of commercial AM fungal biofertilizer to soils containing native, locally adapted AM fungal communities, and demonstrated an urgent need for the establishment of global quality standards for commercial AM fungal inoculum products. We found the best-case scenario of amending native grassland soils containing healthy and diverse AM fungal communities with commercial products was that inoculation was largely ineffective. However, inoculation with several commercially available products resulted in a loss of biomass production, coupled with decreased AM fungal root colonization, indicating that commercial products may de-couple plant–fungal symbiotic relationships. It is critical for restoration practitioners, scientists, and native plant growers to assess the presence of local AM fungal communities before adding unnecessary, or possibly detrimental, AM fungal propagules.

## Figures and Tables

**Table 1:** Functional group, species, and status of common grassland plants selected for commercial arbuscular mycorrhizal (AM) inoculant experiments.

Plant Species	Life Cycle, Provenance
C_4_ Grasses	
*Andropogon gerardii*	Perennial, native
*Bothriochloa ischaemum*	Perennial, non-native, invasive
*Sorghastrum nutans*	perennial, native
C_3_ Grasses	
*Bromus inermis*	Perennial, non-native, invasive
*Elymus canadensis*	perennial, native
Forbs	
*Ratibida columnifera*	Perennial, native
*Salvia azurea*	Perennial, native
Legumes	
*Desmanthus illinoensis*	Perennial, native
*Desmodium canadense*	Perennial, native

**Table 2:** Arbuscular mycorrhizal (AM) fungal species of native field soil and each commercial inoculum product, as listed by product labels.

Inoculum	Arbuscular Mycorrhizal (AM) Fungal Species (According to Labels)
Native field soil	*Acaulospra spinosa, Claroideoglomus claroideum, C. etunicatum, Entrophospora infrequens, Funneliformis mosseae, Glomus heterosporum, G. aggregatum, G. macrocarpum, G. constrictum, Scutellospora calospora,*
A	*Rhizophagus irregularis, C. etunicatum, F. mosseae*
B	*R. irregularis, C. etunicatum, F. mosseae*
C	*A. spinosa, Cetraspora pellucida, C. claroideum, C. lamellosum, E. infrequens, F. mosseae, Racocetra fulgida*
D	*R. irregularis*
E	*R. irregularis, C. etunicatum, F. mosseae*
F	*R. irregularis, C. etunicatum, F. mosseae, G. aggregatum*

**Table 3:** Inoculum pH and nutrient characteristics (g kg^−1^).

Inoculum	pH	Plant-Available N	Plant-Available P
A	6.0 ^b^	108.13 ± 9.53 ^a^	1099.4 ± 36.37 ^a^
B	7.6^a^	14.01 ± 0.54 ^b^	27.72 ± 2.34 ^c^
C	8.1 ^a^	11.27 ± 1.59 ^b^	31.03 ± 4.84 ^c^
D	7.7 ^a^	7.35 ± 0.16 ^b^	302.69 ± 4.07 ^b^
E	7.5 ^a^	3.48 ± 0.11^b^	58.78 ± 3.04 ^c^
F	7.6 ^a^	11.14 ± 0.69 ^b^	36.68 ± 3.22 ^c^

Values within columns that do not share letters are statistically different from one another, with significance assessed at *p* < 0.05.

## Data Availability

The data presented in this study are available upon request from the corresponding author.

## Supplementary Materials

The following supporting information can be downloaded at: https://www.mdpi.com/article/10.3390/plants11172276/s1, Table S1: Raw biomass values; Table S2: Raw arbuscular mycorrhizal (AM) fungal colonization values.
