# Supplementary material for: Effects of Commercial Arbuscular Mycorrhizal Inoculants on Plant Productivity and Intra-Radical Colonization in Native Grassland: Unintentional De-Coupling of a Symbiosis?
Source: Plants (Basel). 2022 Aug 31;11(17):2276. doi: 10.3390/plants11172276 (PMC9460666; doi:10.3390/plants11172276)
Supplement: Supplementary file 1 [file plants-11-02276-s001.zip › plants-1866157-supplementary.pdf]

Table S1. Total biomass [dry weight (mg)] of selected plant species grown in whole soil (control) or in whole soil with added commercial mycorrhizal product (inoc). Within each inoculum treatment (A-F), asterisks denote significant differences (P < 0.05).

	A		B		C		D		E		F	
Species	Control	Inoc	Control	Inoc	Control	Inoc	Control	Inoc	Control	Inoc	Control	Inoc
A. *gerardii*	103*	15	177	304	202	154	198	171	276	260	231*	111
S. nutans	102	380	137	138	163	264	158	151	218	168	203	143
B. ischaemum	161	198	223	308	337	221	275	250	273	225	231	151
E. canadensis	260	200	148	180	202	180	208*	102	155	165	95	88
B. inermis	264	220	81	128	190	215	162	273	175	173	62	140*
D. illinoensis	187	115	139	256	136	202	221	190	193	163	100	130
D. canadense	198	125	205	311	446	429	333	469	192	195	378	279
R. columnifera	110	53	118	100	114	176	89	89	131	126	128	116
S. azurea	386	696*	280	310	532	617	260	291	356	408	405	208

Table S2. Arbuscular mycorrhizal (AM) fungal colonization of selected plant species grown in whole soil (control) or in whole soil with added commercial mycorrhizal product (inoc). Within each inoculum treatment (A-F), asterisks denote significant differences (P < 0.05).

	A		B		C		D		E		F	
Species	Control	Inoc	Control	Inoc	Control	Inoc	Control	Inoc	Control	Inoc	Control	Inoc
A. *gerardii*	24.6	NA	30	36.3	39.3	26	28.3*	15.6	31*	19	32.6	23.3
S. nutans	39.6	22.3	41.6	44	42	40	36*	21	36.6	36	37	43
B. ischaemum	24.6*	8.3	22.3	31.6	21	20.6	22.3	12.3	33.3	17	22.3	22.6
E. canadensis	24.3	15	28	30.6	25	25	24.3*	13.3	23	21.6	23.6	25
B. inermis	10	21	17	26.6	16.6	18.6	19*	12.3	21	19.3	17.3	24
D. illinoensis	36.6*	21.6	47	55	48	43	50.6*	24.3	48.3	44	43.6	46
D. canadense	40.3*	19	47.3	45	45.6	48	42.3*	32	48.6	42.3	46.6	45.3
R. columnifera	18	8	13.3	13.6	11.6	13.6	16.3	7.6	13.6	10.6	17	16
S. azurea	37.6	16.3	39.6	44.6	41	46.3	35.6	21	43.3	43.6	46.6	47.6
